# Protein expression pattern of calcium-responsive transactivator in early postnatal and adult testes

**DOI:** 10.1007/s00418-020-01942-1

**Published:** 2021-01-04

**Authors:** Ana Du, Li Li, Zhaoshuang Jiao, Gaochun Zhu, Ting Peng, He Li

**Affiliations:** 1 Department of Histology and Embryology, School of Basic Medical Sciences, Tongji Medical College, Huazhong University of Science and Technology, Wuhan, 430030 China; 2 School of Biomedical Sciences, LKS Faculty of Medicine, The University of Hong Kong, Hong Kong SAR, China; 3 Department of Anatomy, School of Basic Medicine, Nanchang University, Nanchang, 330006 China; 4 Hubei Key Laboratory of Embryonic Stem Cell Research, Hubei University of Medicine, Shiyan, 442000 China

**Keywords:** CREST, Sertoli cells, Spermatogenic cells, Spermatogenic epithelium, Cell differentiation

## Abstract

Calcium-responsive transactivator (CREST), a nuclear protein highly expressed in postmitotic neurons, is involved in the regulation of cell cycle, differentiation and dendritic development of neuronal cells. Its mRNA has been detected in the testis of adult rat, whilst its protein expression and distribution pattern in the testis remain to be elucidated. In this study, we examined the distribution of CREST in the adult testes of both rats and human as well as the expression pattern of CREST in the testes of postnatal developing rats. In the adult testes of both human and rats, immunohistochemical analysis revealed that CREST was selectively distributed in the mature Sertoli cells but not in the spermatogenic cells. In the testes of postnatal developmental rats, CREST was expressed not only in Sertoli cells but also in the gonocytes and spermatogenic cells at the initial stage of spermatogenic cell differentiation. CREST immunoreactivity continued to increase in Sertoli cells during differentiation, reaching its peak in adulthood. However, CREST immunostaining intensity dramatically decreased as the spermatogenic cells differentiate, disappearing in the post-differentiation stage. Furthermore, Brg1 and p300, two CREST-interacting proteins ubiquitously expressed in the body, are found to be colocalized with CREST in the spermatogenic epithelial cells including Sertoli cells. The unique expression pattern of CREST in developing testis suggests that CREST might play regulatory roles in the differentiation of spermatogenic epithelial cells. The Sertoli cell-specific expression of CREST in the adulthood hints that CREST might be a novel biomarker for the mature Sertoli cells.

**Supplementary Information:**

The online version of this article (10.1007/s00418-020-01942-1) contains supplementary material, which is available to authorized users.

## Introduction

Calcium-responsive transactivator (CREST) is a nuclear protein with a high homology to synovial sarcoma translocation (SYT) proto-oncogene with 54% amino acid identity (Storlazzi et al. 2003; Aizawa et al. 2004). Earlier studies with Northern blot, in situ hybridization, immunohistochemistry and Western blotting have all shown that CREST is enriched in the brain with an exclusive distribution in neurons than the neuroglia (Aizawa et al. 2004; Wu et al. 2007). The characteristic pattern of CREST expression in the developing brain has also been investigated. In the late embryonic rat brain, for instance, CREST is only expressed in the postmitotic neurons of the cortical plate, but not in the proliferating neurons (Aizawa et al. 2004). CREST expression in the rat brain is high at birth and declines substantially throughout early postnatal development, but remains at medium to high levels in the adulthood. Such development-dependent expression of CREST in the nervous system is suggestive of its regulative function on neuronal. Subsequent functional analysis of CREST via gene targeting and differentiation induction reveals that an appropriate level of CREST is important for neuronal differentiation (Aizawa et al. 2004; Wang et al. 2012b). Furthermore, CREST has been found to induce arrest of cell cycle and differentiation of the neurons through switching the chromatin remodeling complex called switch/sucrose nonfermentable (SWI/SNF), also known as Brahma-related gene/Brahma (Brg/Brm)-associated factor (BAF), by replacing its homologous protein SS18 in the complex (Staahl et al. 2013).

In addition to expression in the nervous system, CREST mRNA can also be detected in the peripheral organs of the rats, such as the heart, liver, kidney, pancreas and testis (Aizawa et al. 2004; Men et al. 2013). Direct relation of the increase in expression of CREST in pancreatic β-cells of hyperglycemic rats to high glucose-induced apoptosis suggests multifunction of CREST in non-nervous tissues (Men et al. 2013). Interestingly, *crest* mutant mice often develop infertility (Aizawa et al. 2004) and a series of proteins interacting with CREST are found closely related to the differentiation and maturation of the spermatogenic epithelial cells (Gye et al. 2001; Don and Stelzer 2002; Boussouar et al. 2014; Carre et al. 2018). Histone acetyltransferases p300 and CREB-binding protein (CBP), two of the earliest identified CREST-interacting proteins, regulate the determination and differentiation of testicular tissues as well as the metabolic remodeling in the later stages of spermatogenesis (Gye et al. 2001; Don and Stelzer 2002; Boussouar et al. 2014; Carre et al. 2018). CREB itself, on the other hand, participates in the differentiation of Sertoli cells (Chaudhary and Skinner 2001; Saxlund et al. 2004). Moreover, CREST-containing SWI/SNF complex undergoes a variety of activity changes during the development of spermatogenic epithelium (Kim et al. 2012; Wang et al. 2012a; Menon and Shibata 2019). Transcriptional activator Brg1, a catalytic subunit of the SWI/SNF complex that inhibits CREST-mediated transcription, is preferentially enriched at the promoters of active genes essential for the pluripotency and meiosis of the spermatogonia; deficiency of Brg1 leads to meiotic arrest and abnormal protein expression of the seminiferous epithelium (Kim et al. 2012; Wang et al. 2012a; Serber et al. 2016; Menon and Shibata 2019). All of these studies strongly imply that CREST and the protein–protein interactions it mediates could play a crucial role in testicular development. However, the expression and distribution of CREST at the protein level in mature and developing testis has not been investigated.

In this study, we examined the expression and distribution of CREST in adult rat and human testes, and analyzed the changes of CREST expression in the testicular tissues of the postnatal rats. We show here that CREST expression increases in the Sertoli cells but decreases in the spermatogenic cells with postnatal development, which implies that CREST could be an important factor in regulating early postnatal differentiation of the spermatogenic epithelium.

## Materials and methods

### Reagents

Rabbit polyclonal antibody against CREST was purchased from Proteintech Group (Wuhan, China). Rabbit monoclonal antibody against Wilms’ tumor protein (WT1) was purchased from Abcam (Cambridge, UK). Mouse monoclonal antibody against GATA-4 was purchased from ABclonal Technology (Wuhan, China). Mouse monoclonal antibody against Brg1 and mouse monoclonal antibody against p300 were purchased from Santa Cruz Biotechnology (Dallas, TX, USA). Biotinylated anti-rabbit IgG and avidin–biotin complex (ABC) were purchased from Vector Labs (Burlingame, CA, USA). HRP-conjugated goat-anti-rabbit IgG was purchased from Servicebio (Wuhan, China). Rhodamine Red X-conjugated donkey anti-rabbit IgG, FITC-conjugated donkey anti-rabbit IgG, FITC-conjugated donkey anti-mouse IgG, normal donkey serum (NDS) and normal goat serum (NGS) were purchased from Jackson ImmunoResearch Laboratories. Tyramide signal amplification-Cyanine 3 (TSA Plus CY3) was purchased form PerkinElmer (Waltham, MA, USA). Bovine serum albumin (BSA) was purchased from VWR (Solon, OH, USA). Protein ladder was purchased from Thermo Fisher scientific (Waltham, MA, USA). 6-diamidino-2-phenylindole (DAPI), 3, 3′-diaminobenzidine tetrahydrochloride (DAB), glutathione high capacity magnetic agarose beads, phenylmethylsulfonyl fluoride (PMSF) and protease inhibitor cocktail were purchased from Sigma-Aldrich (Saint Louis, MO, USA). Fusion protein of full-length CREST to glutathione S-transferase (GST) (GST-CREST) was generated in our laboratory according to the standardized protocols as described previously (Li et al. 2003).

### Collection of human and animal testes

Human testes were obtained from two donors (aged 17 and 60, respectively) without testicular disease. Sprague–Dawley rats were housed in a temperature-controlled room with a 12-h light/12-h dark cycle and were given free access to standard food and water. At the designed experimental time points, rats were killed by decapitation under ketamine hydrochloride anesthesia (100 mg/kg; intraperitoneal injection). The testes of the adult humans and rats (6 months old) were fixed in 0.1 M sodium phosphate buffer (PB, pH 7.4) containing 4% paraformaldehyde for 24 h (rats) to 48 h (human), embedded in paraffin, and serially sectioned at a thickness of 2 µm on a microtome (RM2135, Leica, Germany). The testes of the postnatal rats (P1, P5, P7, P9, P10, P14, P22, P34) were fixed in 0.1 M PB containing 4% paraformaldehyde for 12 h, cryoprotected in 0.1 M PB containing 30% sucrose, embedded in OCT, quickly frozen, and then cut into sections of 9 µm on a cryostat (CM1900, Leica, Germany) at − 20 °C. At each developmental time point, testes of at least three rats were collected.

### Immunohistochemistry

The expression of CREST was checked in the adult rat and human testes using immunofluorescence. After deparaffinization and hydration, the testicular sections were subjected to antigen retrieval with 0.01 M sodium citrate buffer (pH 6.0) in a microwave oven for 10 min, permeabilized with 0.3% Triton X-100 in 0.01 M phosphate-buffer saline (PBS; pH 7.4) at room temperature (RT) for 30 min, and blocked with a mixture of 5% NDS and 3% BSA in PBS at RT for 30 min. The sections were incubated with primary antibody against CREST (1:800) overnight at 4 °C, Rhodamine Red X (RRX)-conjugated donkey-anti-rabbit IgG (1:100) was used as a secondary antibody to incubate the sections in the dark at RT for 2 h. Antibodies were diluted with PBS containing 5% NDS and 3% BSA and the sections were rinsed in PBS three times for 10 min each between incubations. After counterstaining with 0.1 µg/ml DAPI in PBS, drying in the air and mounting with 10% glycerol, immunofluorescence observation was performed under a laser scanning confocal microscope.

To detect colocalization of CREST and WT1 (a Sertoli cell marker), double avidin–biotin complex (ABC) staining on adjacent sections and double immunofluorescence labeling on the same section were used. For double ABC staining on adjacent sections, the sections of the adult rat testes were deparaffinized, antigen retrieved, and then sequentially treated with PBS containing 0.3% Triton X-100, 1% hydrogen peroxide in PBS, a mixture of 5% NGS and 3% BSA in PBS for 30 min each to reduce endogenous peroxidase activity and nonspecific binding of antibody. Thereafter, the adjacent sections were incubated with primary antibody anti-CREST (1:300) or anti-WT1 (1:250) overnight at 4 °C, followed by incubation in biotinylated goat-anti-rabbit IgG (1:200) at RT for 2 h and in ABC complex (1:100) at RT for 2 h. Primary and secondary antibodies and ABC complex were diluted with PBS containing 5% NGS and 3% BSA. Between incubations, the sections were washed in PBS three times for 10 min each. Finally, the immunoreactive products of CREST or WT1 were visualized by incubating with 0.02% DAB and 0.005% hydrogen peroxide in 0.05 M Tris–HCl (pH 7.6) for 10–15 min at RT. After being dehydrated, cleared and cover slipped, the sections were examined under a light microscope. For double immunofluorescence staining of CREST and WT1 on the same sections, multiple immunolabeling with antibodies from the same host species in combination with tyramide signal amplification (TSA) was performed according to the previously published protocols (Stack et al. 2014; Buchwalow et al. 2018). Briefly, the deparaffinized, hydrated and antigen-retrieved paraffin sections of the adult rat and human testes were permeabilized and blocked as described above. Thereafter, sections were incubated with CREST antibody (1:3000) overnight at 4 °C, followed by incubation of HRP-conjugated goat-anti-rabbit IgG (1:200) for 50 min at RT and TSA Plus Cy3 (1:100) in amplification diluent (0.02% H_2_O_2_ in PBS) for 10 min at RT. Next, sections were processed antigen retrieval again as described above for elution of the previous primary/secondary antibody complex (CREST/IgG-HRP antibody complex). After washing in PBS, sections were incubated with WT1 antibody (1:200) for 1 h at RT, followed by incubation with FITC-conjugated donkey-anti-rabbit IgG (1:500) for 30 min at RT. Finally, the sections were counterstained with 0.1 µg/ml DAPI for nuclear staining, mounted in 10% glycerol for observation under the laser scanning confocal microscope.

For double immunofluorescent labeling of CREST and GATA-4, CREST and Brg1 or CREST and p300 in the postnatal rat testes, standard immunostaining procedures were performed as described previously (Liao et al. 2010). Briefly, the frozen sections were incubated in a mixture of anti-CREST and anti-GATA-4 antibodies (1:200), anti-CREST and anti-Brg1 (1:200) or anti-CREST and anti-p300 (1:200) at 4 °C overnight after permeabilized with Triton X-100 and blocked in NDS and BSA, followed by incubation with a mixture of RRX-conjugated donkey anti-Rabbit IgG and FITC-conjugated donkey anti-mouse IgG (1:500) at RT for 2 h. Finally, the sections were counterstained and examined as described above.

### Specificity of the primary antibodies

The specificity of the anti-CREST antibody (Proteintech, 12439-1-AP; immunogen: C-terminal amino acids 47-396 of human CREST) utilized in the present research has been tested on the human and mouse tissues by co-immunoprecipitation and immunohistochemistry analyses (Chesi et al. 2013). The specificity of anti-WT1 antibody (Abcam, ab89901), anti-GATA-4 antibody (Abclonal, A3600), anti-Brg1 antibody (Santa Cruz, sc-17796) and anti-p300 antibody (Santa Cruz, sc-48343) has been tested on mouse tissues by immunofluorescence analysis (anti-WT1, anti-GATA-4 and anti-Brg1 antibodies) (Del Monte-Nieto et al. 2018; Morohoshi et al. 2019; Zhang et al. 2019) and Western blot analysis (anti-WT1, anti-GATA-4, anti-Brg1 and anti-p300 antibodies) (Bao et al. 2018; Dou et al. 2018; Shorstova et al. 2019; Zhang et al. 2019). In addition, the specificity of the anti-CREST antibody was tested in the present study by Western blotting (Fig. S1), substitution (primary antibody against CREST was replaced by normal rabbit serum) and absorption (primary antibody against CREST was preabsorbed with excess GST full-length CREST fusion protein) experiments (Fig. S2). For the specificity of CREST and WT1 immunofluorescence staining on the same section (TSA assay), normal rabbit serum was used to replace antibody anti-CREST or anti-WT1 (Fig. S3).

### Western blot: specificity of anti-CREST antibody

The brain, spinal cord and testis from 8- to 10-week-old rats were homogenized and lysed in buffer [50 mM Tris–HCl (pH8.0), 1% TritonX-100, 150 mM NaCl, 1 mM EDTA, 1 mM PMSF] with 1 × protease inhibitor cocktails, centrifuged at 12,000 × *g* for 15 min at 4 °C. The supernatant was separated by 12% SDS-PAGE and transferred onto nitrocellulose membrane (GE Healthcare Life Sciences, Marlborough, MA, USA). Primary antibody to CREST (1:2000) was tested according to the protocol described before (Liao et al. 2010). Western blot analysis revealed a band of ~ 55 kDa in the protein preparation of testis, the same as that detected in the brain and spinal cord protein preparations (theoretical molecular weight of CREST is 55 kDa) (Fig. S1), confirmed the specificity of the primary antibody anti-CREST.

### Microscopy

The ABC-stained preparations were examined on a bright field light microscope (Olympus BX5, Tokyo, Japan) and the images were taken with a CCD camera (Nikon DXM1200, Tokyo, Japan; image size: 1360 × 1024 pixel; 24 bit). Immunofluorescence observation was performed using a laser scanning confocal microscope (Olympus Fluoview FV1000, Tokyo, Japan). The laser diodes used were 559 nm for excitation of RRX, 550 nm for excitation of Cy3, 488 nm for excitation of FITC and 405 nm for excitation of DAPI. The fluorescence image was recorded with PMT (synchronizing with scanning mirrors) (image size: 1024 × 1024 pixel, Bits/Pixel: 12 bits).

### Quantification of CREST expression and statistical analysis

The immunofluorescence intensity of nuclear CREST that represents CREST expression level in the gonocyte, GATA-4-positive (GATA-4^+^) immature Sertoli cell, GATA-4-negative (GATA-4^−^) spermatogenic cell or WT1-positive (WT1^+^) mature Sertoli cell was measured using the Image-Pro Plus 6.0 software. The immunofluorescence intensity of CREST was the average signal density of each selected nucleus, which was calibrated by subtracting the signal intensity of back ground. For each of the four kinds of cells, more than 100 cells from three to five rats of each postnatal age point were measured. Data were presented as mean ± SD with nuclear CREST immunofluorescence intensity. For statistical analyses of data, GraphPad Prism 6 Software (GraphPad Inc.) was used. CREST expression levels were analyzed by use of unpaired, two-tailed Student’s *t* test. *P* values of less than 0.05 was considered statistically significant.

## Results

### CREST is highly and exclusively expressed in the mature Sertoli cells of adult testis

**Fig. 1:**
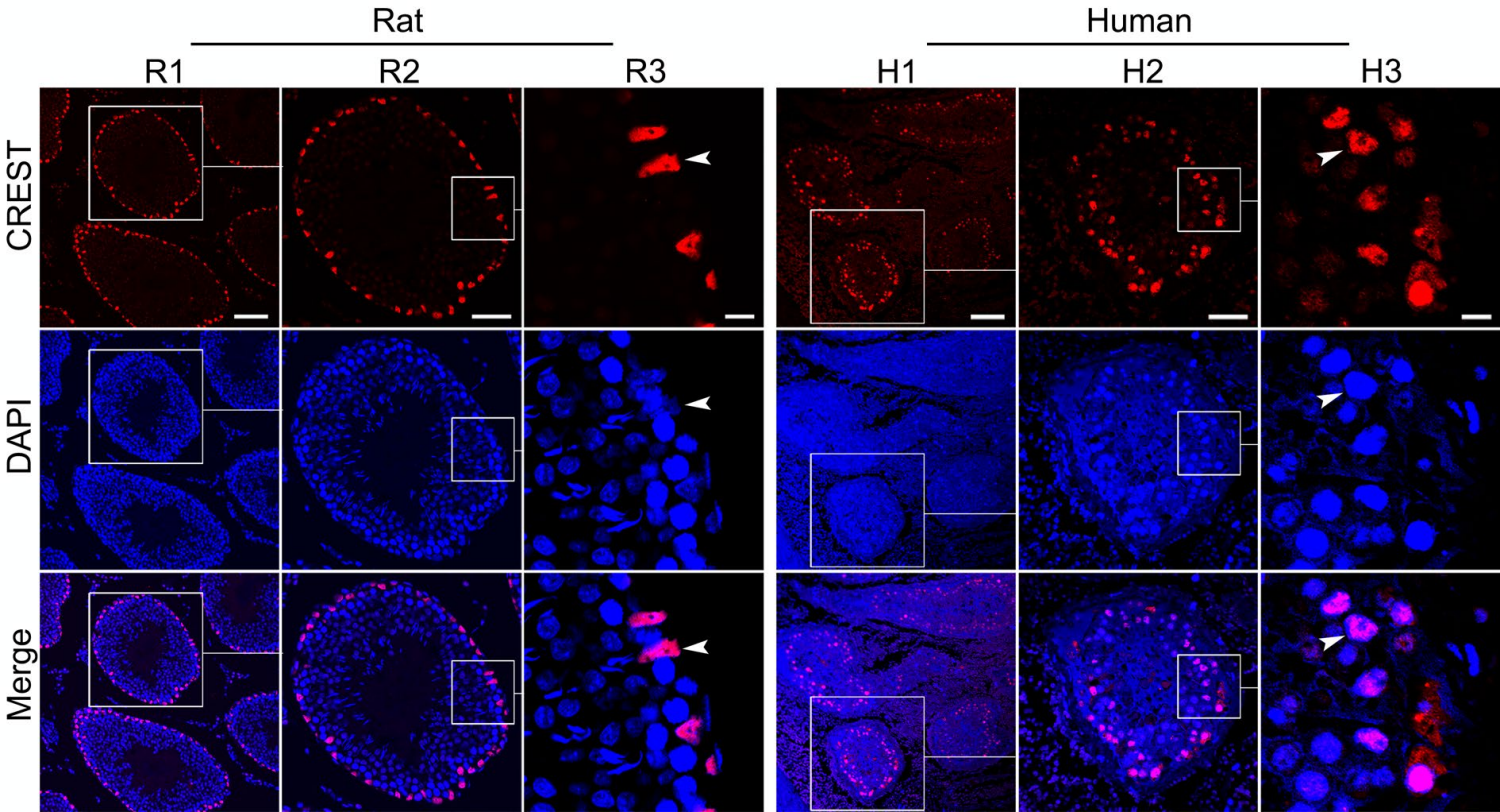
CREST protein expression in the testes of a 6-month-old rat (Rat) and a 17-year-old human (Human). CREST is labeled by RRX (red) and nuclei are stained with DAPI (blue). R2 and H2 are magnified images of the boxed areas in R1 and H1, respectively, R3 and H3 are magnified images of the boxed areas in R2 and H2, respectively. Merge images show that CREST immunoreactivity is in the nuclei of the cells at the basal region of the seminiferous tubules, assumed Sertoli cells. Arrowheads indicate the CREST-positive nuclei that are triangular in shape with indentations and a prominent central nucleolus. Scale bars: 100 μm in R1 and H1, 50 μm in R2 and H2, and 10 μm in R3 and H3

To clarify whether CREST is expressed in the adult testis, single immunofluorescence staining for CREST protein was carried out on the adult testicular tissue sections of the rat and human. It was found that CREST was highly expressed and exclusively distributed in the nuclei of cells at the basal region of the seminiferous tubules of the adult rats (6 months old) (Fig. 1R1–R3). The nuclei of the mature Sertoli cells are triangular in shape with indentations and a prominent central nucleolus (Nistal et al. 2011). Our results strongly suggest a unique localization of CREST in mature Sertoli cells. In the adult human testes, the expression and localization of CREST were similar to those in the adult rat testes (Fig. 1H1–H3). There was no difference of CREST localization in the seminiferous tubules between 17-year-old and 60-year-old testes, although the CREST immunoreactivity in the older testis was somewhat weaker than that in the younger one (data not shown).

**Fig. 2:**
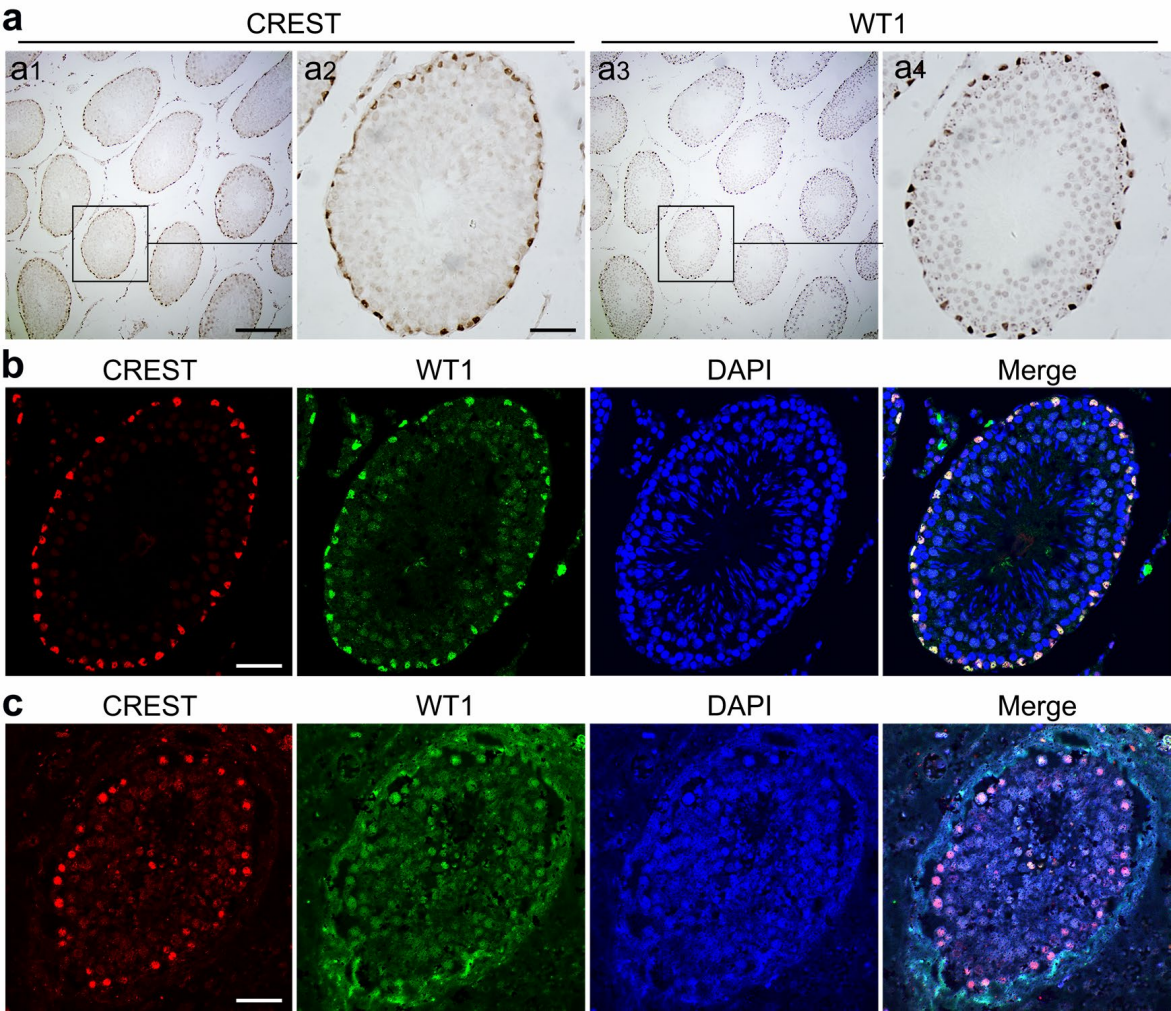
Immunohistochemical analysis for localization of CREST and WT1 in the adult seminiferous tubules of both rat and human. **a** ABC staining of CREST (a1, a2) and WT1 (a3, a4) in adjacent serial sections of the adult rat testis (6 months old) shows localization of CREST and WT1. Higher magnification image of the boxed areas in a1 and a3 are shown in a2, a4. TSA immunofluorescence double staining on the same sections of the 6-month-old adult rat testis (**b**) and the 17-year-old adult human testis (**c**); CREST is labeled by CY3 (red), WT1 by FITC (green), and nuclei were stained by DAPI (blue). Merge images in **b** and **c** show the colocalization of CREST and WT1 in the nuclei. Scale bars: 200 μm in a1 and a3, and 50 μm in a2, a4 (**b**, **c**)

Subsequently, WT1 (Wilms’ tumor protein), a widely used specific marker of mature Sertoli cells (Segunda et al. 2019), was selected to analyze if CREST is selectively expressed in the mature Sertoli cells. The adjacent section ABC double staining on 2-µm-thick serial sections of the adult rat testis showed that CREST was colocalized with WT1 in the Sertoli cells of the adult rat testis (Fig. 2a). To further validate the colocalization of CREST and WT1 in the Sertoli cells, we performed double immunofluorescence staining. The double immunofluorescent labeling based on tyramine signal amplification (TSA) technique confirmed that CREST and WT1 were well colocalized in the Sertoli cells of both adult rats (Fig. 2b) and human (Fig. 2c) testes.

### Expression of CREST in the Sertoli cells increases with postnatal development of the rats

**Fig. 3:**
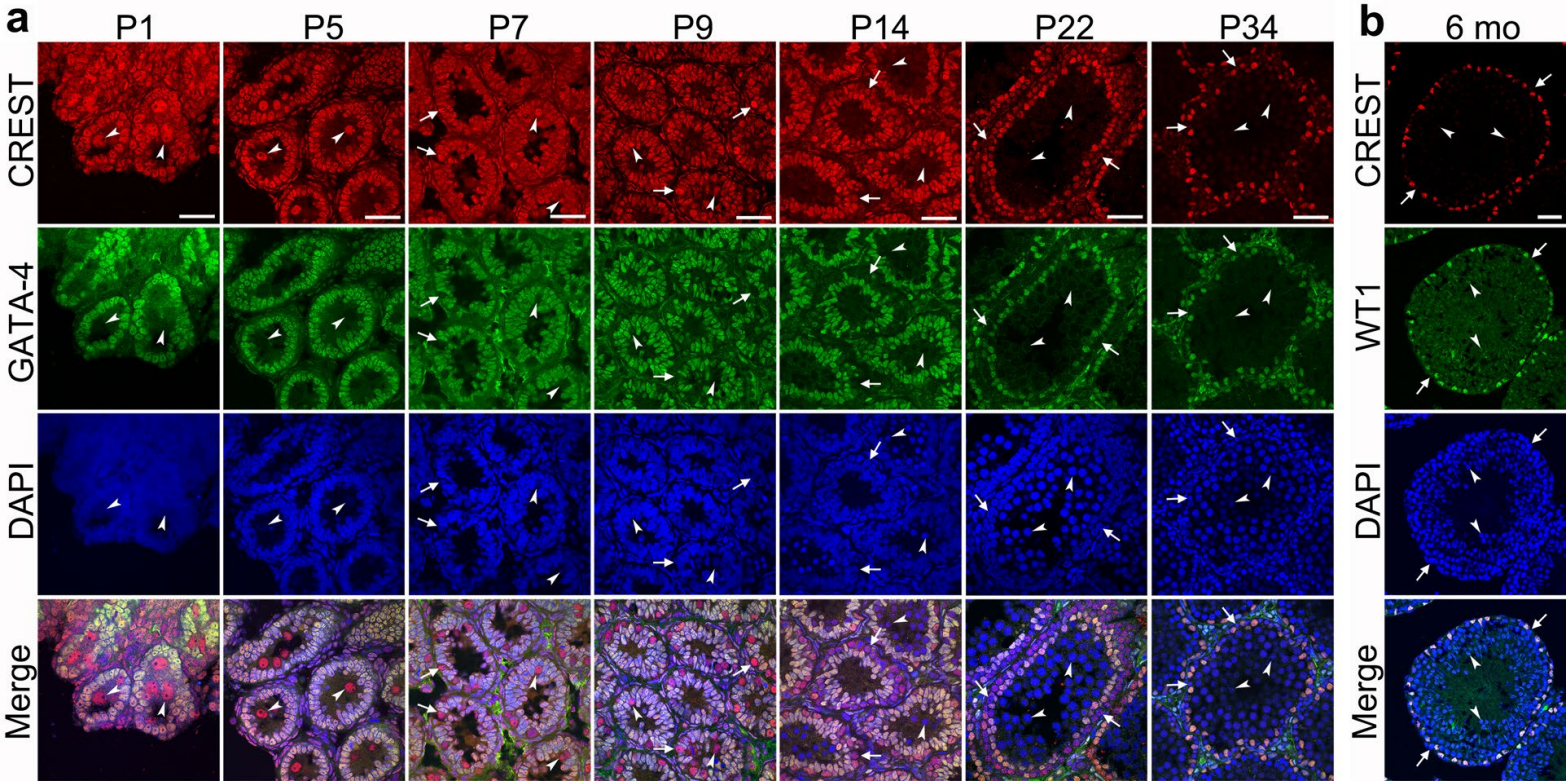
Development**-**dependent expression of CREST in the seminiferous tubules of the postnatal developing and adult rats. Immunofluorescence double staining for the colocalization of CREST and GATA-4 (**a**) in the rat testes at P1, P5, P7, P9, P14, P22, P34 and colocalization of CREST and WT1 (**b**) in the testis at 6 months old (6 mo). CREST is labeled by RRX (red) (**a**) and by CY3 (red) (**b**), GATA-4 (**a**) and WT1 (**b**) by FITC (green), and nuclei are counterstained with DAPI (blue). Arrowheads indicate the spermatogenic cells at the adluminal portion, arrows indicating the spermatogenic cells at the basal portion. Scale bars: 40 μm

To see if CREST might play a role in development of Sertoli cells as it does in development of neurons (Aizawa et al. 2004; Staahl et al. 2013), the expression pattern of CREST in the rat testis at different postnatal stages (P1, P5, P7, P9, P14, P22, P34, 6 month) was examined using double immunofluorescent labeling in which GATA-4 was used as a marker for immature Sertoli cells while WT1 as a marker for mature Sertoli cells (Viger et al. 1998; Walker 2003; Segunda et al. 2019). At early postnatal stages (P1–P14), CREST immunofluorescent intensity in GATA-4^+^ Sertoli cells was strong, and gradually increasing with developmental stages. With the gradual maturation of Sertoli cells from P22 to P34 during which GATA-4 immunoreactivity gradually decreased in the Sertoli cells, the immunofluorescence signal of CREST continued to increase. Up to the adulthood (6 months old, 6 months), CREST immunofluorescence signal reached a peak in mature Sertoli cells which strongly expressed WT1 (Figs. 3, 5a).

### Expression of CREST decreases dramatically in early postnatal spermatogenic cells and ceases when reaching adulthood

**Fig. 4:**
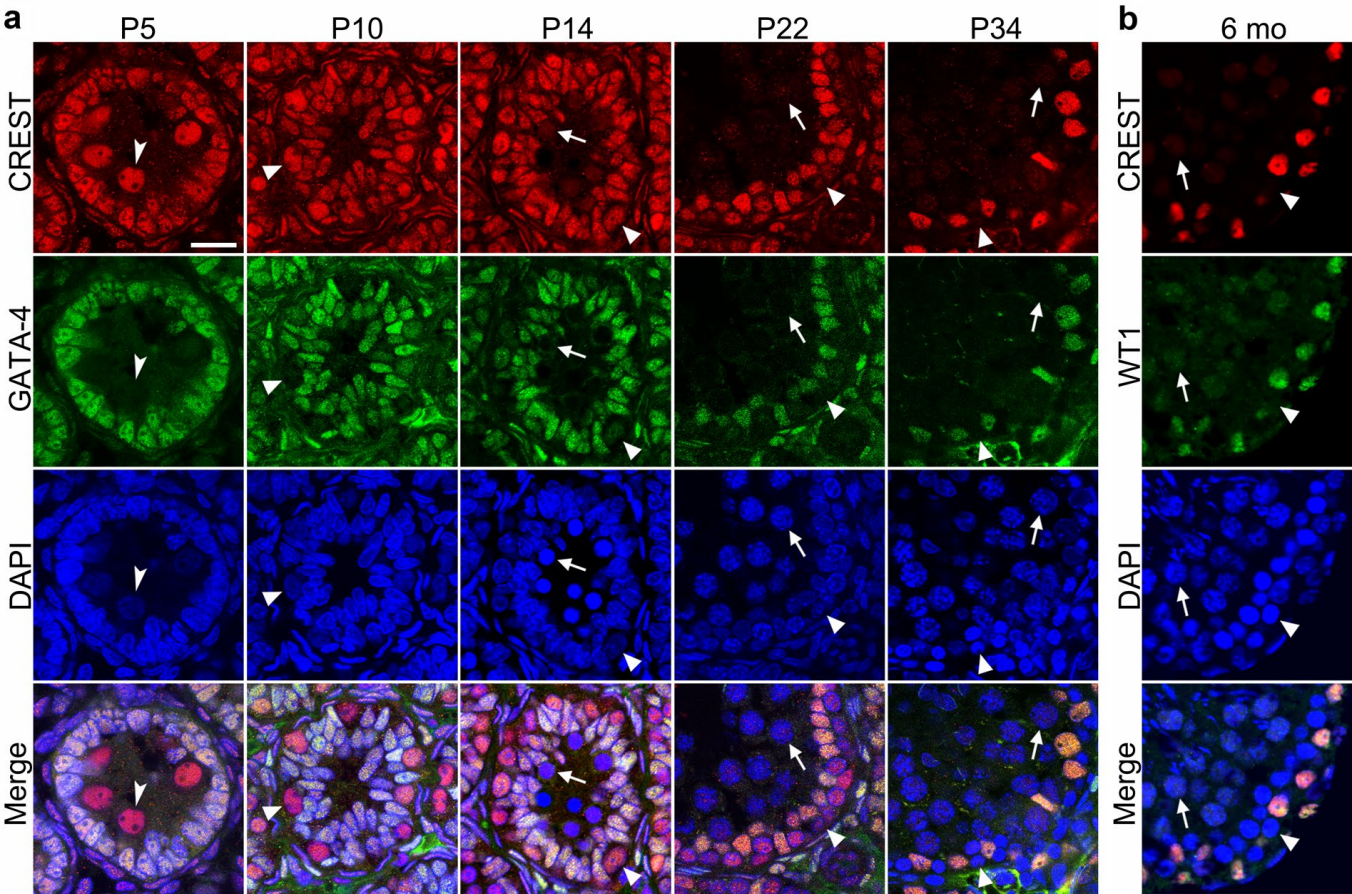
Differential expression of CREST in the Sertoli cells and spermatogenic cells of the postnatal developing and adult rat testes. **a** Immunofluorescence double staining for the colocalization of CREST and GATA-4. **b** Immunofluorescence double staining for the colocalization of CREST and WT1 showing that CREST is labeled by RRX (red) (**a**) or CY3 (red) (**b**), GATA-4 (**a**) and WT1 (**b**) by FITC (green), and nuclei by DAPI (blue). Arrowheads indicate gonocytes, Arrows and filled triangles indicate spermatogenic cells in the adluminal and basal portions, respectively. Scale bar: 20 µm

**Fig. 5:**
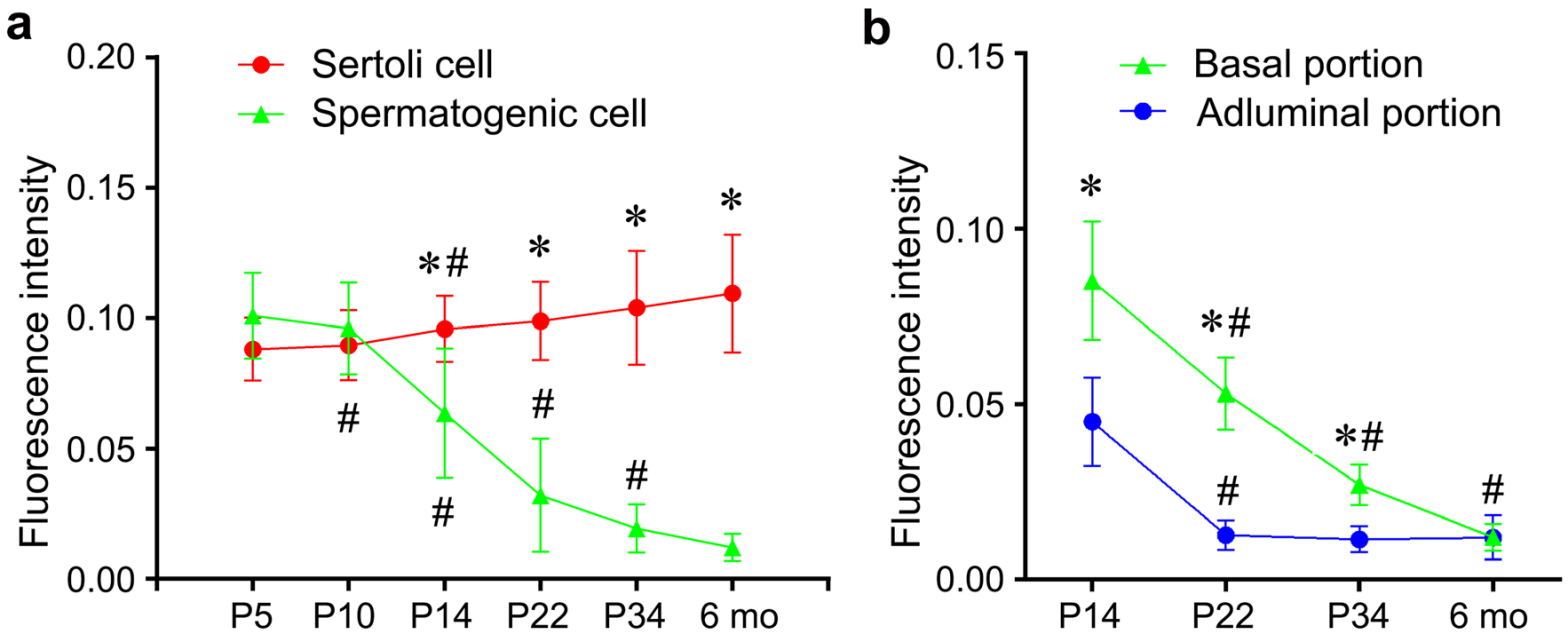
Quantifications of nuclear CREST immunofluorescence intensity in developing seminiferous epithelium of postnatal rat testes. **a** Statistical analysis of the immunofluorescence intensity of the nuclear CREST in the Sertoli cells and spermatogenic cells. **b** Statistical analysis of the immunofluorescence intensity of the colocalization nuclear CREST in the spermatogenic cells located in the basal and adluminal compartments. The immunofluorescence intensity of CREST was quantified using Image-Pro Plus 6.0 software. “Asterisk” and “ash” indicate significance at *P* < 0.05. “Astersik” indicates a comparison between two types or portions of cells (Sertoli cell vs Spermatogenic cells or basal portion *vs* abluminal portion) at the same time point; “ash” indicates a comparison with the previous time point of the same type cells or the cells in the same portion

On the seminiferous tubule sections of the young rats, especially in the rats at early postnatal stages (P1 to P14), examination using double immunofluorescent labeling of CREST with GATA-4 detected that CREST was expressed not only in the GATA-4^+^ immature Sertoli cells but also in the GATA-4^−^ developing spermatogenic cells (Figs. 3, 4). From P1 to P5, CREST immunoreactivity could be detected in the GATA-4^−^ gonocytes located in the adluminal portion of seminiferous tubules, and was even stronger than that in the immature Sertoli cells (Figs. 3, 4, 5a). Around P7, strong CREST immunoreactive signal could also be detected in gonocytes migrating from the adluminal portion to the basal portion (Figs. 3, 4). However, at P14, the expression of CREST began to decrease in the spermatogenic cells (Figs. 3, 4), especially in spermatocytes at the adluminal portion (Fig. 5b). At P22, CREST-positive signal could hardly be detected in the adluminal spermatogenic cells and dramatically decreased in the basal spermatogenic cells as well (Figs. 4, 5b). At P34, CREST was no longer expressed in spermatogenic cells, but only in mature Sertoli cells (Figs. 4, 5).

### CREST is colocalized with Brg1 and p300 in the spermatogenic epithelium of postnatal developing and adult rats

**Fig. 6:**
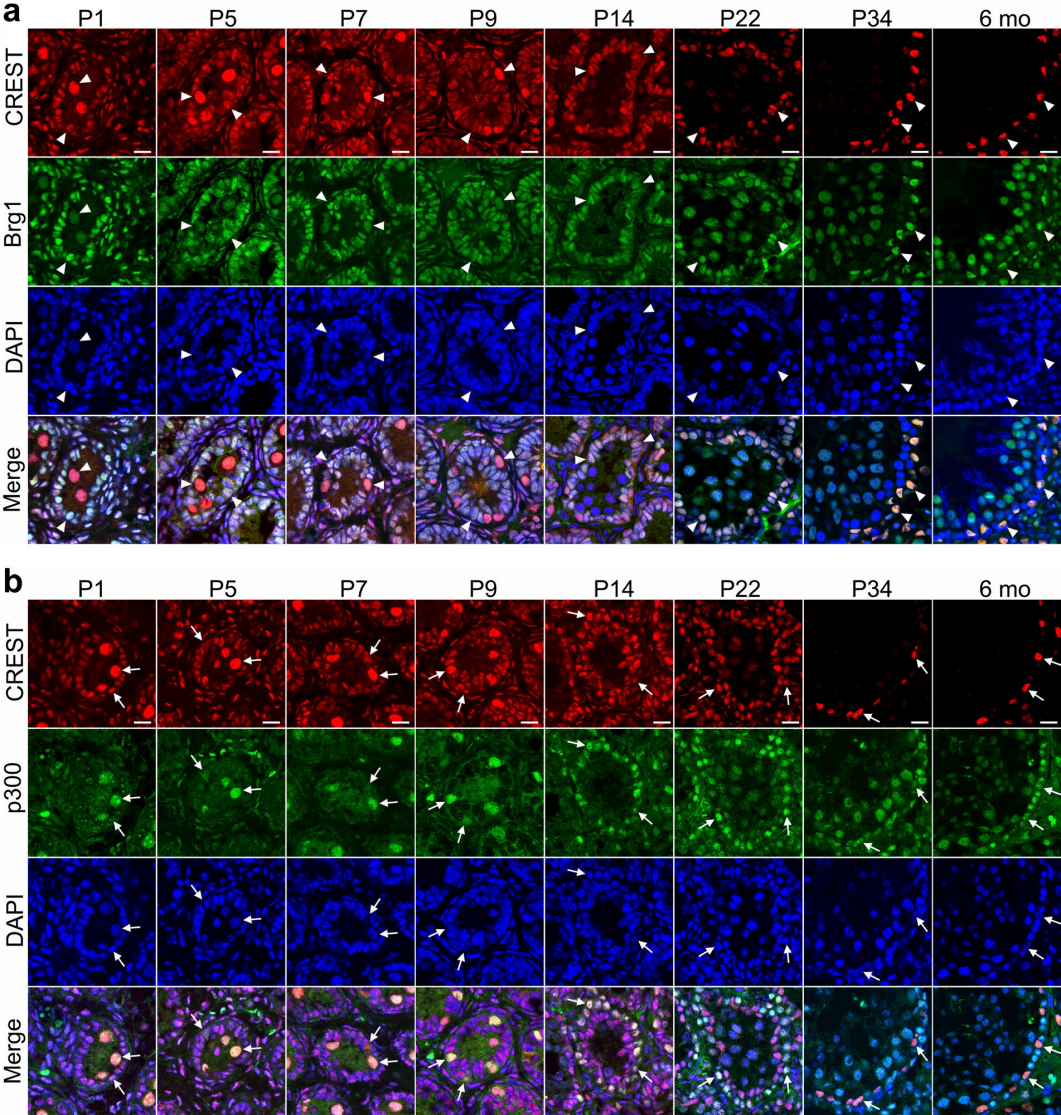
The expression of CREST, Brg1 and p300 in the seminiferous tubules of the postnatal developing and adult rats**.** Immunofluorescence double staining for the localization of CREST and Brg1 (**a**) or CREST and p300 (**b**) in the rat testes at P1, P5, P7, P9, P14, P22, P34 and 6 months old (6 mo). CREST is labeled by RRX (red) (**a**, **b**), Brg1 (**a**) and p300 (**b**) by FITC (green), and nuclei are counterstained with DAPI (blue). Filled triangles indicate the colocalization of CREST with Brg1 (**a**). Arrows indicate the colocalization of CREST with p300 (**b**). Scale bars: 20 μm

To address if CREST also regulate the differentiation of spermatogenic epithelial cells through interacting with Brg1 and p300, we further examined if CREST protein is co-expressed with Brg1 and p300 in the postnatal seminiferous tubules by using double immunofluorescence staining. As expected, Brg1 and p300 were found to be widely expressed in the rat spermatogenic epithelium from neonatal to adult stages, though Brg1 expression was low in the spermatogenic cells and p300 expression low in the Sertoli cells. Furthermore, at early postnatal stage both spermatogenic cells and Sertoli cells were double labeled for CREST–Brg1 and CREST–p300, but at adult stage, only Sertoli cells were double labeled (Fig. 6 and Fig. S4).

## Discussion

Previous study with Northern blot and in situ hybridization analysis has indicated that CREST mRNA is widely expressed in various peripheral organs and tissues including the testis (Aizawa et al. 2004); whereas, the expression of CREST at the protein level has only been detected in the brain and pancreas with Western blot and /or immunohistochemistry (Aizawa et al. 2004; Men et al. 2013). In the present study, we demonstrated for the first time CREST protein expression in the rat and human testis with immunohistochemical staining. Moreover, in the developing rat testis, CREST immunoreactivity was found to increase with postnatal development of Sertoli cells expressing GATA-4 and decrease with the differentiation of spermatogenic cells that were negative for GATA-4. In the adult rat testis, on the other hand, CREST protein was selectively localized in the WT1-positive Sertoli cells while disappeared in the spermatogenic cells.

The role of CREST in the regulation of neuronal differentiated development has been implied through the age-dependent expression change of CREST in the mouse brain, N2a neuroblastoma cells. An increase in the expression of differentiation- and maturation-associated genes, as well as cell cycle arrest, was found to correlate with high CREST levels in the N2a cells, while targeted disruptions of the *crest* gene resulted in defects in mouse neurite development (Aizawa et al. 2004; Wang et al. 2012b; Staahl et al. 2013). The development-related change in the expression and localization of CREST in the rat spermatogenic epithelium, as observed in the present study, hints that CREST might also be involved in the regulation of the differentiation of Sertoli cells and spermatogenic cells in the testis.

The period for differential expression of CREST in the spermatogenic epithelium coincides with the differentiation stage of the spermatogenic epithelial cells. In the spermatogenic epithelia of rats on the first 10 days after birth, the immature Sertoli cells are located in the basal compartment, while the spermatogenic cells in stage of gonocyte are located in the adluminal compartment (Pariante et al. 2016). Around P7, the Sertoli cells start to assist gonocytes transiting from the adluminal region toward the basal region (Pariante et al. 2016; Gautam et al. 2018; Venditti and Minucci 2019). That is to say, the cells in the spermatogenic epithelium of rats only changes in location but not in the state of differentiation from P1 to P10. It is at this stage that the expression level and distribution of CREST in both Sertoli cells and spermatogenic cells do not change significantly. Around P13 is a critical time point for the differentiation of rat seminiferous epithelium cells (Bittman 2016): the gonocytes begin to differentiate into spermatogonia around P11, and type A spermatogonia begin to proliferate in mitosis with initiation of early meiosis of type B spermatogonia and appearing of spermatocytes in the lumen of seminiferous tubule at about P14 (Pariante et al. 2016); on the other hand, the Sertoli cells stop proliferating and begin to mature about 14 days after birth (Gautam et al. 2018). It is noteworthy that, during this period (from P10 to P14), CREST immunoreactivity increases significantly in the Sertoli cells but decreases rapidly in the differentiated spermatogenic cells, especially in newly formed spermatocytes in the adluminal portion of the seminiferous tubule. Interestingly, expression level of CBP and P300, two CREST-interacting proteins, are high in the spermatogonia and early spermatocytes but low in the cells after initiation of meiosis (Boussouar et al. 2014), which is very similar to the expression of CREST in the spermatogenic cells at these developmental stages. Furthermore, the unique expression change of CREST and CREST-interacting proteins during spermatogenic epithelial differentiation is corresponding to the arrest of Sertoli cell cycle and the initiation of spermatogenic cell division. In view of that CREST induces cell cycle arrest and differentiation of N2a cell (Staahl et al. 2013), it is, therefore, reasonable that the CREST expression change around P14 could be a key step for the initiation of cell cycle change and differentiation of the spermatogenic epithelium.

As one of the calcium ion responsive transactivators, CREST has no domain that can directly bind to DNA, so it cannot directly activate gene transcription (Aizawa et al. 2004; Jefferis et al. 2004). However, it can mediate protein–protein interaction in the way as an adaptor, thus affecting the assembly, activation and functional transformation of many protein complexes (Storlazzi et al. 2003; Aizawa et al. 2004; Qiu and Ghosh 2008; Staahl et al. 2013). It has been proved that substitution of CREST for its homologous protein SS18 in switch/sucrose nonfermentable (SWI/SNF) or neural progenitor Brg/Brm-associated factor (npBAF) complex, one of the combinatorial assembled chromatin regulatory complexes, is essential for differentiation of neural progenitors into neurons (Qiu and Ghosh 2008; Staahl et al. 2013). The npBAF complex incorporated with SS18 is required for neural stem cell self-renewal. Neuronal differentiation induction increases expression of CREST, which results in replacement of SS18 in npBAF complex by CREST and then leads to a series of changes in subunits of the complex and functional switching of npBAF to nBAF. The nBAF complex incorporated with CREST loses its promoting effect on cell proliferation, neuronal differentiation is, thus, initiated (Staahl et al. 2013). Cell differentiation of postnatal spermatogenic epithelium is critical to normal testicular development in mammals, and this process is strictly regulated by gene transcription and activity of combinatorial assembly chromatin regulatory complexes (Johnston et al. 2008; Lui and Cheng 2012; Menon and Shibata 2019). A variety of CREST-interacting proteins, such as p300 and CBP, and the components of CREST-containing nBAF complex take key roles not only in neurogenesis and tumorigenesis (Romero and Sanchez-Cespedes 2014; Pulice and Kadoch 2016), but also in spermatogenic epithelial development and spermatogenesis (Kim et al. 2012; Menon and Shibata 2019). For example, knockout of Brg1 leads to abnormal protein expression in spermatogenic epithelium and arrest of meiosis (Menon and Shibata 2019). In addition, many components of SWI/SNF complex, such as RUSH and BRD7, are closely related to the development of spermatogenic epithelium. Rush is highly expressed in the immature Sertoli cells of rabbit testis after birth and before puberty (Rendon et al. 2000), and the deficiency of BRD7 results in impaired spermatogenesis and male infertility in mice (Wang et al. 2016). In view of these, it is likely that CREST in the spermatogenic epithelium also changes the components and function of BAF complex through its development-dependent expression change, resulting in regulation of maturation and differentiation of Sertoli cells and spermatogenic cells in a manner similar to that in neurons. Supporting of this presumption is our double immunostaining results that CREST is well colocalized with two main CREST-interacting proteins, Brg1 and p300, non-exclusively in the early postnatally developing spermatogenic cells and Sertoli cells, as well as exclusively in the differentiated adult Sertoli cells.

The most striking finding of our study is that CREST is persistently expressed at a high level in the Sertoli cells but not, or very weakly if any, in the spermatogenic cells of the adult rat testis. The selective expression of CREST in the adult Sertoli cells means that CREST can be used as a specific marker of mature Sertoli cells. From this, it is also implicated that CREST might possess unique function in mature Sertoli cells. In fact, there are some similarities between mature neurons and Sertoli cells: both are out of cell cycle, terminally differentiated (Chaudhary et al. 2005; Hobert 2011; Hayrabedyan et al. 2012); the cytoplasm of both cells develop or grow continuously due to their respective functions, for example, the dendrites of mature neurons branch because of the establishment of new neural networks (Tien and Kerschensteiner 2018), and the cytoplasm of mature Sertoli cells also continue to grow because of movement and maturation of spermatogenic cells (Vogl et al. 2008; Upadhyay et al. 2012). The normal expression of CREST is the basis of cell cycle control and dendrite development of mature neurons. Therefore, we believe that the high expression of CREST in mature Sertoli cells may also be closely related to terminal differentiation maintenance, cell cycle stability and continuous cytoplasm development.

Owing to the difficulty of collecting human neonatal testes, the expression and distribution of CREST in postnatal developing testis were only detected in the rats. Our further work is to collect human testicular tissues at different postnatal stages to clarify whether the testicular CREST expression pattern in the postnatal developing human individuals is similar to that in postnatal developing rats.

## Electronic supplementary material

Below is the link to the electronic supplementary material.

Supplementary file1 (TIF 457 KB) Fig. S1 Western blot analysis for the specificity of antibody anti-CREST utilized in the present study. Antibody anti-CREST recognizes a major band in the testis, the molecular weight of which is the same as that of the band in the brain or spinal cord, closed to the theoretical molecular weight (55 kDa) of CREST

Supplementary file2 (TIF 40504 KB) Fig. S2 Control experiments of immunofluorescence analyzing the specificity of anti-CREST primary antibody in the sections of a 6-month-old rat testis. (a1–a3) Immunofluorescence staining with antibody anti-CREST showing CREST-positive labeling with RRX (red). (b1–b3) Substitution experiment with replacement of primary antibody anti-CREST by normal rabbit serum showing CREST-negative labeling. (c1–c3) Absorption experiment with absorption of primary antibody anti-CREST by recombinant full-length CREST showing CREST-negative labeling. Nuclei are labeled with DAPI (blue) in a–c. Scale bar: 100 μm

Supplementary file3 (TIF 53740 KB) Fig. S3 Control experiments of TSA double immunofluorescence on the same section analyzing the specificity of two primary antibodies in the sections of a 6-month-old rat testis. (a1–a4) TSA immunofluorescence double staining of CREST and WT1 using rabbit polyclonal antibody anti-CREST and rabbit monoclonal antibody anti-WT1 showing that CREST is labeled by CY3 (red), WT1 by FITC (green). (b1–b4) Substitution experiment of TSA double immunofluorescence staining of CREST and WT1 using rabbit monoclonal antibody anti-WT1 and normal rabbit serum (replacing antibody anti-CREST) showing CREST-negative labeling and WT1-positive labeling with FITC (green). (c1–c4) Substitution experiment of TSA double immunofluorescence staining of CREST and WT1 using rabbit polyclonal antibody anti-CREST and normal rabbit serum (replacing antibody anti-WT1) showing CREST-positive labeling by CY3 (red) and WT1-negative labeling. Nuclei are labeled with DAPI (blue) in a–c. Scale bar: 100 μm

Supplementary file4 (TIF 43694 KB) Fig. S4 The expression of CREST, Brg1 and p300 in the seminiferous tubules of the postnatal developing and adult rats. Double immunofluorescence staining for the colocalization of CREST and Brg1 (a) or CREST and p300 (b) in the rat testes at P1, P5, P7, P9, P14, P22, P34 and 6 months old (6 mo). CREST is labeled by RRX (red) (a and b), Brg1 (a) and p300 (b) by FITC (green), and nuclei are counterstained with DAPI (blue). Scale bars: 50 μm
