# Supplementary figures and images for: Protein expression pattern of calcium-responsive transactivator in early postnatal and adult testes
Source: Histochem Cell Biol. 2021 Jan 4;155(4):491–502. doi: 10.1007/s00418-020-01942-1 (PMC8062385; doi:10.1007/s00418-020-01942-1)

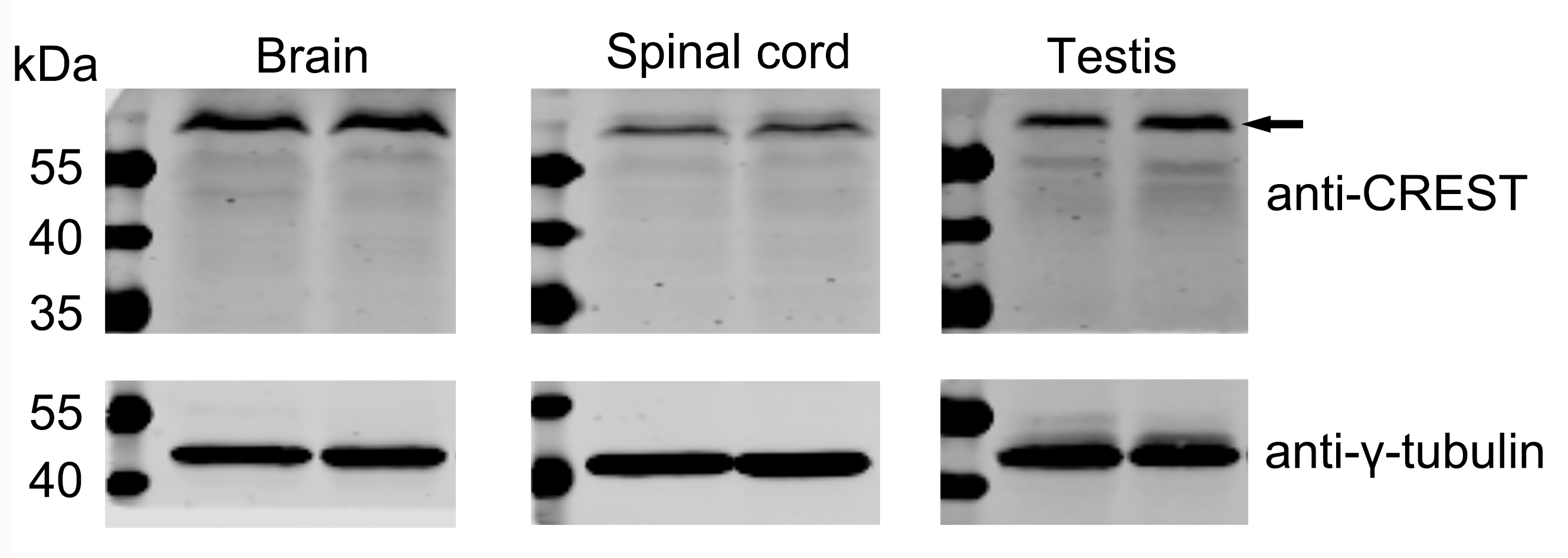

Supplement: Supplementary file 1 — Supplementary file1 (TIF 457 KB) Fig. S1 Western blot analysis for the specificity of antibody anti-CREST utilized in the present study. Antibody anti-CREST recognizes a major band in the testis, the molecular weight of which is the same as that of the band in the brain or spinal cord, closed to the theoretical molecular weight (55 kDa) of CREST [file 418_2020_1942_MOESM1_ESM.tif]

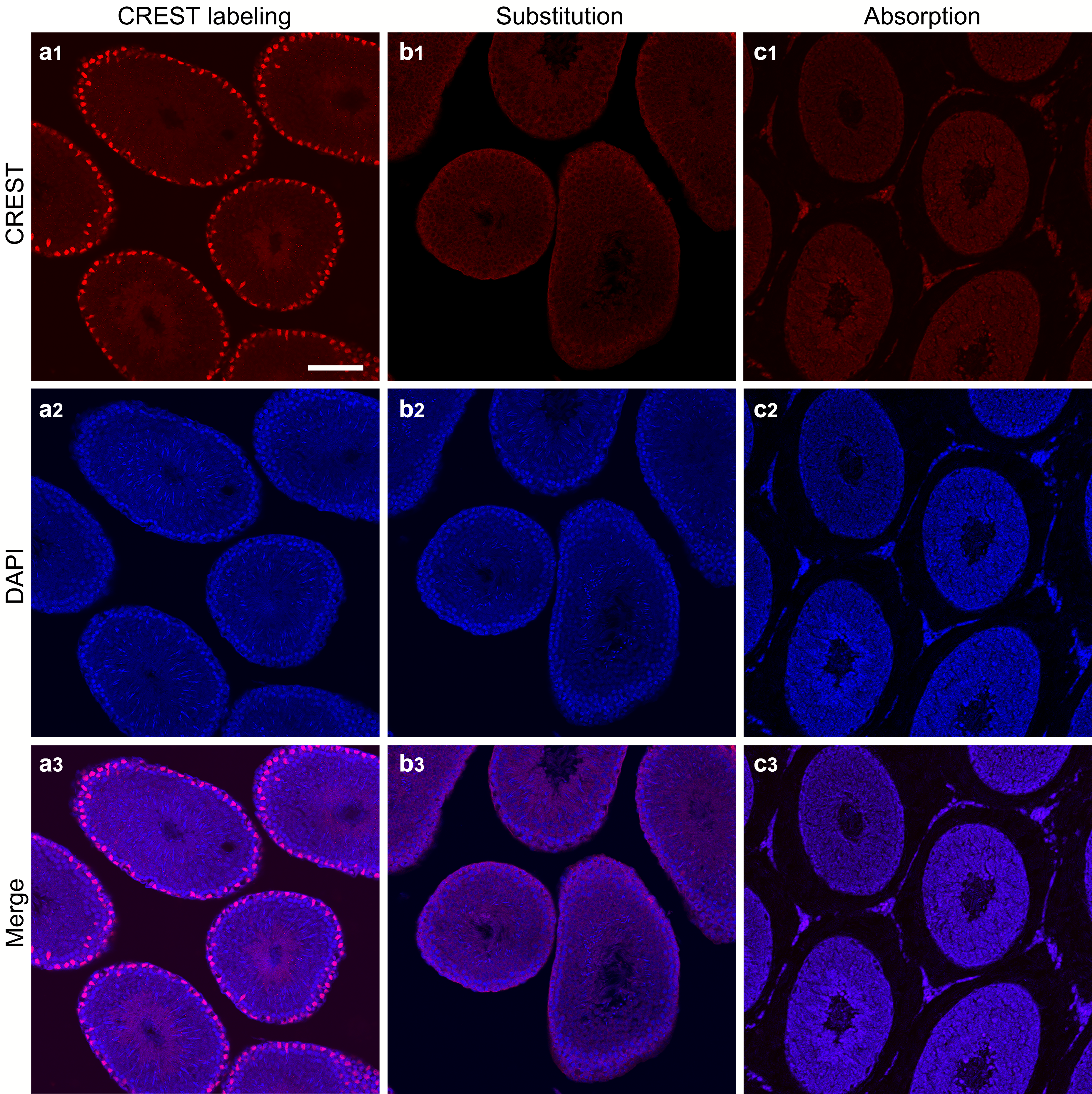

Supplement: Supplementary file 2 — Supplementary file2 (TIF 40504 KB) Fig. S2 Control experiments of immunofluorescence analyzing the specificity of anti-CREST primary antibody in the sections of a 6-month-old rat testis. (a1–a3) Immunofluorescence staining with antibody anti-CREST showing CREST-positive labeling with RRX (red). (b1–b3) Substitution experiment with replacement of primary antibody anti-CREST by normal rabbit serum showing CREST-negative labeling. (c1–c3) Absorption experiment with absorption of primary antibody anti-CREST by recombinant full-length CREST showing CREST-negative labeling. Nuclei are labeled with DAPI (blue) in a–c. Scale bar: 100 μm [file 418_2020_1942_MOESM2_ESM.tif]

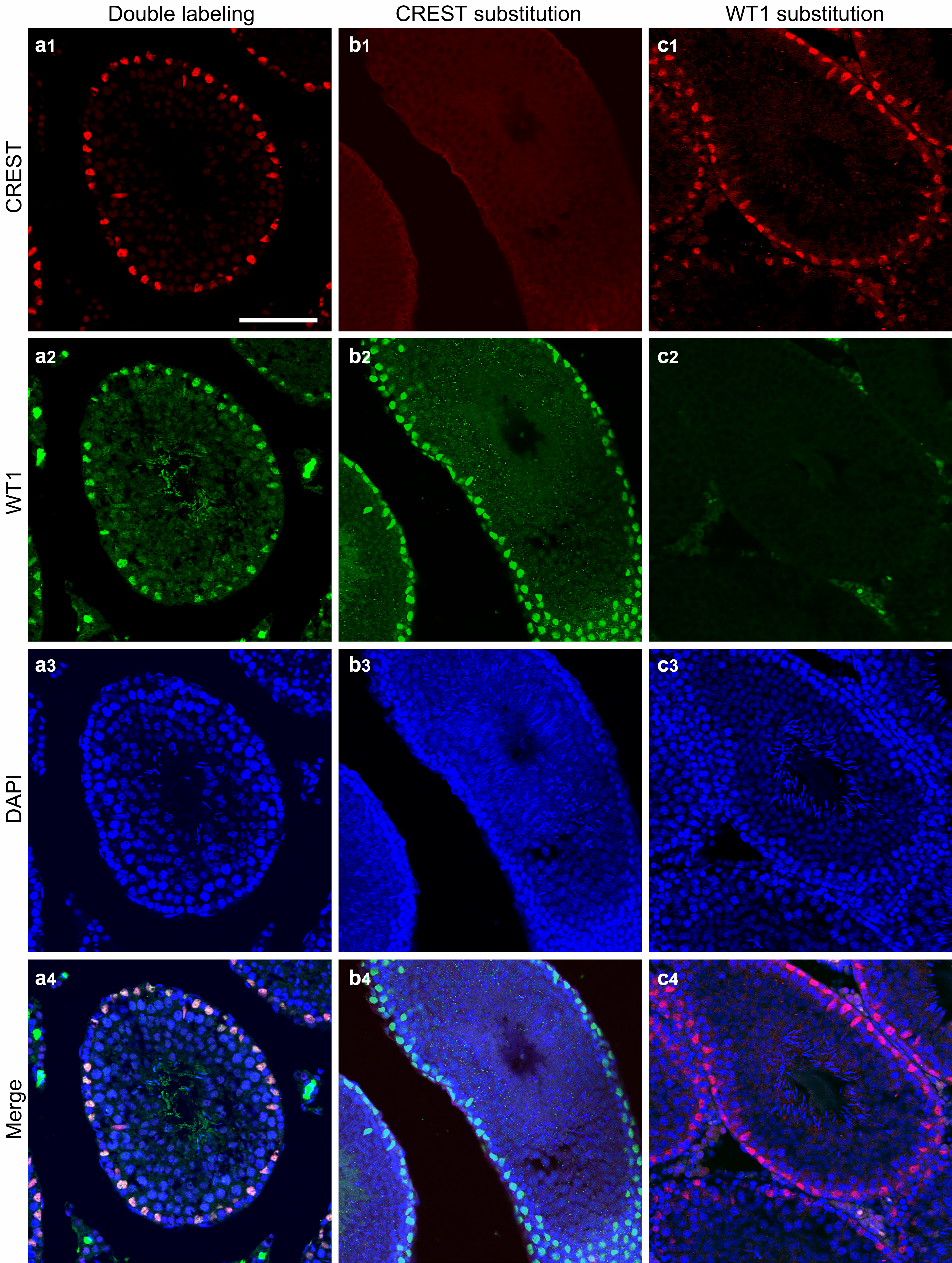

Supplement: Supplementary file 3 — Supplementary file3 (TIF 53740 KB) Fig. S3 Control experiments of TSA double immunofluorescence on the same section analyzing the specificity of two primary antibodies in the sections of a 6-month-old rat testis. (a1–a4) TSA immunofluorescence double staining of CREST and WT1 using rabbit polyclonal antibody anti-CREST and rabbit monoclonal antibody anti-WT1 showing that CREST is labeled by CY3 (red), WT1 by FITC (green). (b1–b4) Substitution experiment of TSA double immunofluorescence staining of CREST and WT1 using rabbit monoclonal antibody anti-WT1 and normal rabbit serum (replacing antibody anti-CREST) showing CREST-negative labeling and WT1-positive labeling with FITC (green). (c1–c4) Substitution experiment of TSA double immunofluorescence staining of CREST and WT1 using rabbit polyclonal antibody anti-CREST and normal rabbit serum (replacing antibody anti-WT1) showing CREST-positive labeling by CY3 (red) and WT1-negative labeling. Nuclei are labeled with DAPI (blue) in a–c. Scale bar: 100 μm [file 418_2020_1942_MOESM3_ESM.tif]

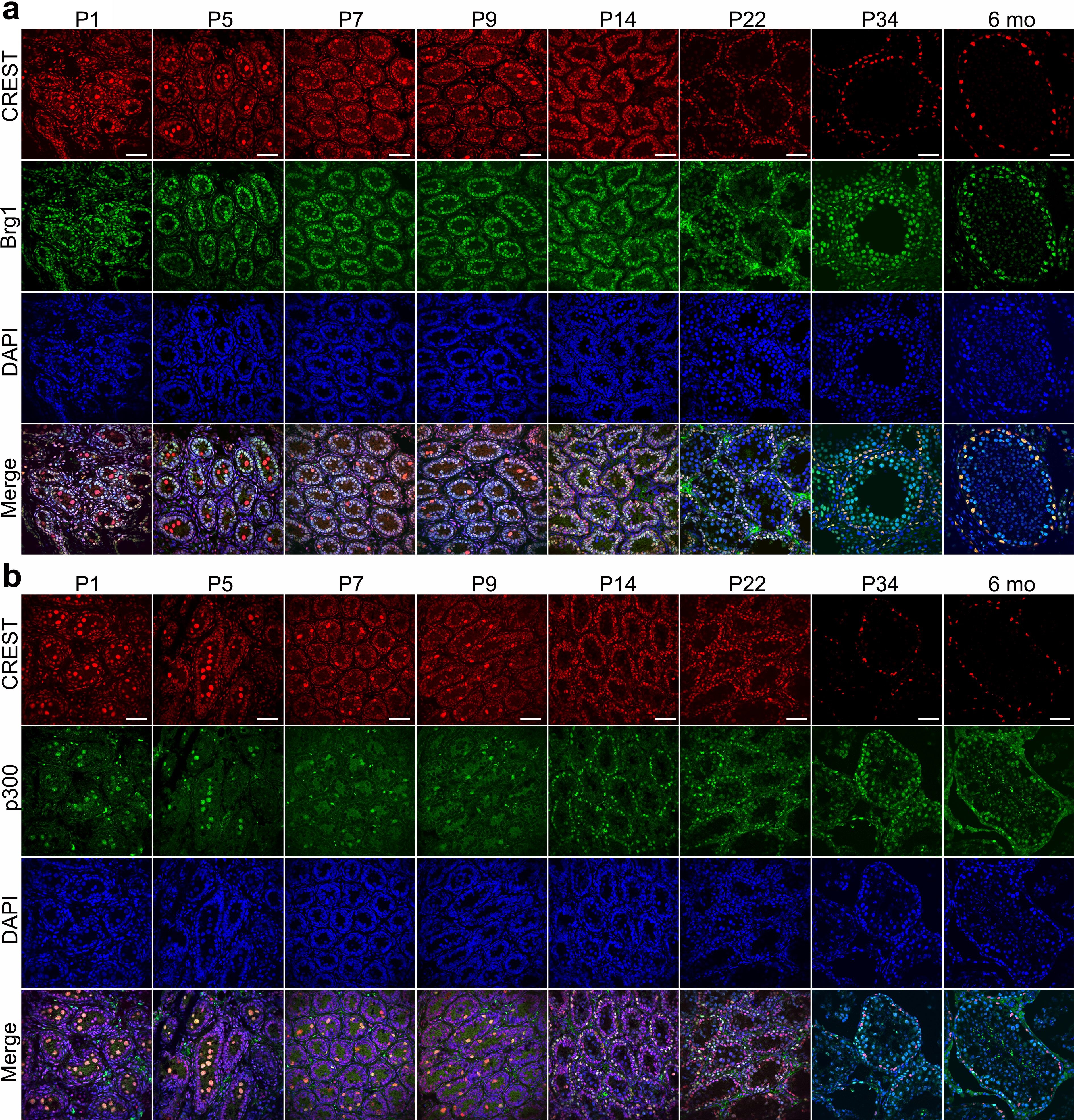

Supplement: Supplementary file 4 — Supplementary file4 (TIF 43694 KB) Fig. S4 The expression of CREST, Brg1 and p300 in the seminiferous tubules of the postnatal developing and adult rats. Double immunofluorescence staining for the colocalization of CREST and Brg1 (a) or CREST and p300 (b) in the rat testes at P1, P5, P7, P9, P14, P22, P34 and 6 months old (6 mo). CREST is labeled by RRX (red) (a and b), Brg1 (a) and p300 (b) by FITC (green), and nuclei are counterstained with DAPI (blue). Scale bars: 50 μm [file 418_2020_1942_MOESM4_ESM.tif]
